# Social inequalities and full vaccination coverage at 24 months – 2017-2018 live birth cohort: National Vaccination Coverage Survey, 2020

**DOI:** 10.1590/1980-549720250049

**Published:** 2025-11-03

**Authors:** Rita Barradas Barata, Ana Paula França, Ione Aquemi Guibu, Carla Magda Alan Domingues, Maria da Gloria Teixeira, José Cássio de Moraes

**Affiliations:** I Faculdade de Ciências Médicas da Santa Casa de São Paulo, Department of Collective Health – São Paulo (SP), Brazil.; II Consultant – Brasília (DF), Brazil.; III Universidade Federal da Bahia, Institute of Collective Health – Salvador (BA), Brazil.

**Keywords:** Vaccination coverage, Socioeconomic factors, Cohort studies, Brazil

## Abstract

**Objective:**

To analyze social inequalities in vaccination coverage for the full immunization schedule with valid doses at 24 months of age, according to socioeconomic strata and family, maternal, and child characteristics.

**Methods:**

This is a retrospective 2017–2018 live birth cohort in capitals, the Federal District, and 12 Brazilian cities, recruited in 2020–2021. Participants were followed up from birth using the vaccination cards, and there were no losses to follow-up. Guardians were interviewed and vaccination cards were photographed. Urban census tracts of the 39 cities were grouped into four strata by cluster analysis of average income, income >20 minimum wage, % literate individuals. The same number of census tracts were drawn from each stratum, and children from cohorts of interest were recruited until sample size was complete. The coverage of the full immunization schedule for each child was calculated considering only valid doses (date and interval between doses). Descriptive, stratified analysis, and Poisson regression were performed using the STATA 17.0 survey module.

**Results:**

A total of 37,801 children were included. Vaccination coverage was below 50% in all strata, with strata A and B as the lowest. The probability of having a full immunization schedule was lower from the second-born child onward, in families with intra-household crowding, and difficulties in accessing health services. Exclusive use of public services was associated with higher full coverage.

**Conclusion:**

Vaccination coverage was lower in strata with better standards of living (A/B). Vaccination coverage by family, maternal, and individual factors vary between strata and it is not possible to establish a common pattern.

## INTRODUCTION

The Brazilian National Immunization Program (*Programa Nacional de Imunizações* – PNI) was created in 1973 to provide universal access to a set of vaccines to control vaccine-preventable diseases and reduce infant mortality rates. Free and universal access to immunization throughout the country was only enabled, however, after the establishment of the Brazilian Unified Health System (SUS) in 1990 and subsequent expansion of the network of primary health care centers. Despite assurance of universal access to vaccines, vaccination coverage is uneven depending on the type of vaccine, region, city, socioeconomic strata, and other characteristics related to child, mother, and family^1,2,3^.

Authors of a National Survey conducted for the cohort of live births in 2005 in Brazilian capital cities and the Federal District, already pointed to social inequalities in coverage in some of the cities; and lower coverage in social strata with better standards of living in some of them or lower coverage in strata with worse standards of living, depicting the existing heterogeneity^3^.

Studies on vaccine coverage rarely analyze socioeconomic differences by neighborhood area using valid doses, being limited to the administered doses. In this study, we aim at filling these gaps.

The main objective of this study was to analyze the social inequalities in vaccination coverage, full immunization schedule, that is, valid doses of all vaccines of the National Vaccination Calendar up to 24 months of age, administered to children born in 2017 or 2018, according to socioeconomic strata of the residence area and family, maternal, and child characteristics associated with vaccination coverage.

## METHODS

The data used in this article are part of the National Vaccination Coverage Survey funded by the Brazilian Ministry of Health, aiming to assess full-schedule vaccination coverage at 24 months, according to socioeconomic strata and to identify factors associated with incomplete vaccination or non-vaccination^2^.

The study design was a retrospective cohort. Children born in 2017–2018, aged >24 months were included, thus presenting the outcome, with no loss to follow-up. Each child was followed up by using the vaccination card, from the date of birth until the age of 24 months. The cohort design allows to calculate valid doses administered at the recommended ages and with the correct intervals between doses, from birth to 24 months of age, for each child in the sample, and thus obtaining the full vaccination coverage with valid doses (cumulative incidence). In Chart 1, the full immunization schedule and the criteria for defining valid doses are shown. The methods used in the survey were described in more detail in a previous publication^2^. Children without a vaccination card were considered unvaccinated after the negative search for registration in the PNI information system.

**Chart 1:** Vaccines of the full immunization schedule up to 24 months of age and criteria for valid doses.

Vaccine	Valid dose
BCG	administered in any month
Hepatitis B	administered in any month
Pentavalent (DPT+Hib+HepB) 1st dose	42 days of life or more
Pentavalent (DPT+Hib+HepB) 2nd dose	at least 30 days after the 1st dose
Pentavalent (DPT+Hib+HepB) 3rd dose	at least 30 days after the 2nd dose
IPV 1st dose	42 days of life or more
IPV 2nd dose	at least 30 days after the 1st dose
IPV 3rd dose	at least 30 days after the 2nd dose
Rotavirus 1st dose	45 to 180 days of life
Rotavirus 2nd dose	at least 30 days after the 1st dose, up to 180 days
MenC 1st dose	42 days of life or more
MenC 2nd dose	at least 30 days after the 1st dose
Pneumo 1st dose	42 days of life or more
Pneumo 2nd dose	at least 30 days after the 1st dose
Measles, mumps and rubella 1st dose	365 days of life or more
Measles, mumps and rubella 2nd dose	at least 30 days after the 1st dose
Hepatitis A	365 days of life or more
Chickenpox	452 days of life or more
MenC booster	365 days of life or more
Pneumo booster	365 days of life or more
OPV	365 days of life and three previous IPV doses
DPT	365 days of life or more

BCG: Bacillus Calmette–Guérin vaccine; DPT: diphtheria, pertussis and tetanus vaccine; Hib: *Haemophilus influenzae* type b bacteria vaccine; HepB: hepatitis B vaccine; IPV: inactivated poliovirus vaccine; MenC: meningococcal group C bacteria vaccine; Pneumo: pneumococcal vaccine; OPV: oral polio vaccine.

Socioeconomic strata were defined by cluster analysis, considering all urban census tracts — in each capital, the Federal District, and in 12 cities with over 100 thousand inhabitants not located in the metropolitan region, selected in different states. The stratification variables were: average household income, proportion of literate guardians, and proportion of guardians with monthly income greater than 20 minimum wages. The data were obtained from the 2010 Demographic Census of the Brazilian Institute of Geography and Statistics (IBGE)^4^, the last available for the country at the beginning of the research.

Stratum A corresponds to the cluster of census tracts with the best socioeconomic indicators, followed by strata B, C, and D, all representing the average standards of living of the inhabitants residing in the census tracts that compose them.

The same number of clusters of census tracts were drawn, grouped by the estimated number of live births in 2017–2018, in each stratum. Children were searched through the addresses registered in the Brazilian Live Birth Information System (*Sistema de Informações sobre Nascidos Vivos* – Sinasc) and by active search in the drew areas. Sample losses were due to the non-location of children from cohorts in the drew census tracts, considering that reverse sampling was used to avoid refusals. In the sample, there was a 6% loss, although it was differential for the strata, being greater in stratum A^2^.

The children’s guardians were interviewed to obtain family, maternal, and child’s data, and the vaccination cards were photographed. The information was later incorporated into the database after careful reading by trained professionals.

Family characteristics were: consumption level established by the criteria of the Brazilian Association of Research Companies (*Associação Brasileira de Empresas de Pesquisa*)^5^, being beneficiaries of the *Bolsa Familia* cash transfer program, grandmothers living in the same household, monthly family income, and household crowding (more than two residents per bedroom). Maternal characteristics were divided into: age, having a partner, paid work, self-reported race/skin color (IBGE), and level of education. In turn, the child characteristics were as follows: race/skin color (IBGE), sex, birth order, enrollment in day care or nursery, and having a vaccination card.

In addition to these characteristics, the following was included: use of private services for at least one vaccine, guardians’ decision to vaccinate, difficulties in accessing services, and operational difficulties related to the vaccination program (lack of inputs and absence/attitudes of professionals).

The analyses were performed in the STATA 17.0 program using the survey module for complex samples, with weighting for sample losses and population calibration. Full vaccination coverage with valid doses and 95% confidence interval (95%CI) in each stratum were calculated, and effect measures in the comparison between strata were obtained by Poisson regression considering Stratum A as a reference. Variables exclusively associated with the strata were tested for interaction, and the possible confounding variables were also analyzed. Variables associated with exposure and/or the outcome, which did not intervene between each other, remained in the final adjustment model. The backward stepwise procedure was used in Poisson regression, taking as reference the null model.

For two of the intervening variables, income and level of education, statistically significant effect was only observed among the strata in the higher-value classes, namely level of education equal to 16 years and over and family income of BRL 8 thousand or over. The stratified analysis is presented.

The project was approved by the Ethics Committee on Research Involving Human Beings of Irmandade da Santa Casa de São Paulo (Opinion 4.380.019), and the participants signed informed consent forms.

### Data availability statement

The dataset supporting the findings of this study is not publicly available.

## RESULTS

The study sample comprised 37,801 children born in 2017–2018, distributed among socioeconomic strata, namely: 8,333 in stratum A; 9,418 in stratum B; 9,992 in stratum C; and 10,058 in stratum D. Of these, 12.93% did not have a vaccination card in stratum A; 11.86%, in stratum B; 4.80%, in stratum C; and 7.94%, in stratum D.

In Table 1, we describe the composition of the sample by socioeconomic strata in relation to family, maternal, and child characteristics. In stratum A, the following characteristics predominate: families with high and moderate levels of consumption, less than 10% of families live together with grandmothers, family income of BRL 8 thousand or over, most use private services for vaccination, and approximately 1/5 report difficulties with the immunization program. Mothers are predominantly white, aged 35 years and over, almost 90% have a partner, and 73% work. Children are predominantly white, more than half are enrolled in nurseries or day cares and are the firstborn.

**Table 1:** Family, maternal, and child characteristics in each socioeconomic stratum of the residence area. National Vaccination Coverage Survey, 2020.

Variables	Stratum A	Stratum B	Stratum C	Stratum D
**Family characteristics**				
**Level of consumption**				
High	20.8	8.9	3	0.4
Moderate	50.5	41	31.4	8.7
Low	17.6	31.4	36.5	38.9
Very low	11.1	18.7	29.1	52
*Bolsa Família*	7.3	13.9	20.5	34.5
**Grandmother living in the household**	22.4	24.1	27.4	30.4
**Family income (BRL)**				
<1,000.00	7	15.5	22.3	41.1
1,000.00 to 2,999.00	15.5	27.9	39.3	46.7
3,000.00 to 7,999.00	30.7	32.8	27.1	10.8
≥8,000.00	46.8	23.8	11.3	1.4
**Household crowding**	3.6	5.3	8.5	14.6
**Private services**	56.3	43.4	26.8	9
**Decision to vaccinate (all vaccines)**	97.7	96.7	97.8	97.3
**Difficulties in access**	5.7	7	5.4	8.2
**Difficulties with the program**	20.5	23.0	24.7	23.6
**Maternal characteristics**				
**Mother’s skin color**				
White	60.2	57.7	52.5	37.6
Black	6.7	7.1	8.3	17.1
Mixed-race	31.8	32.4	37.6	43.6
Other (Asian and Indigenous)	1.3	2.8	1.6	1.7
**Maternal age (years)**				
<20	0.8	2.1	1.6	3.1
20 to 34	37.7	42.5	54.2	62.7
35 and over	61.5	55.4	44.2	34.2
**Have a partner**	88.9	83.3	78.4	71.4
**Mother’s paid work**	72.6	63.6	58.1	47.9
**Child characteristics**				
**Child’s skin color**				
White	69.7	62.9	59.3	45.2
Black	2.6	4.4	4.7	10.8
Mixed-race	26.5	31.5	35.1	42.8
Other (Asian and Indigenous)	1.2	1.2	0.9	1.2
**Nursery or day care**	56.9	52.9	48.6	48.5
**Birth order**				
Firstborn	52.1	54.9	51.6	43.9
Second-born	35.4	30.7	32.7	29.8
Third-born	8.9	10.1	10.9	15.1
Fourth-born and so on	3.6	4.3	4.8	11.2

In stratum B, the main differences regarding stratum A are in the slightly-lower levels of consumption and income, in addition to the less frequent use of private services. In stratum C, families have moderate to low levels of consumption, about 20% are beneficiaries of the *Bolsa Familia* program, 28% live together with grandmothers, just over 25% use private vaccination services and report problems with the vaccination program. Most mothers are white, aged 20 to 34 years, about 78% have a partner, and most have paid work. Children are mostly white, but less than half are enrolled in day cares or nurseries, and half are the firstborn.

In stratum D, more than half of the families have very low levels of consumption, 34% are beneficiaries of *Bolsa Família*, about 30% live together with grandmothers, 15% have household crowding, and ¼ report problems with the vaccination program. Mothers are predominantly mixed-race, aged between 20 and 34 years, 70% have a partner, and less than half have paid work. Children are white or mixed-race, less than half are enrolled in day cares or nurseries, and they are mainly firstborn and second-born children.

With regard to the intention to vaccinate, almost all guardians stated that they wanted their children to receive all vaccines, regardless of the socioeconomic stratum.

In Table 2, we show vaccination coverage for the full immunization schedule at 24 months of age, in each socioeconomic stratum, according to family, maternal, and child characteristics. For families living in socioeconomic stratum A, we observed lower coverage at high or moderate levels of consumption, income of BRL 8 thousand or over, use of private vaccination services, white mothers aged <20 or <35 years or over, those who have a partner, level of education of 16 years or over, and white children. However, as some poor families also live in the stratum, we observed lower coverage among those without the right to the *Bolsa Família* income and with maternal education of up to eight years.

**Table 2:** Vaccination coverage by the full immunization schedule at 24 months of age according to family, maternal, and child characteristics in each socioeconomic stratum of the residence area. National Vaccination Coverage Survey, 2020.

Variables	Stratum A	Stratum B	Stratum C	Stratum D
**Family characteristics**				
**Level of consumption**				
High	16.5 (9.9–26.4)	15.8 (7.5–30.1)	18.9 (11–30.5)	47 (13.2–83.7)
Moderate	25.6 (20.5– 31.4)	26.9 (20.8–34)	37.6 (32.1–43.5)	47.5 (40.1–55)
Low	51.4 (42.4–60.3)	45.4 (38.7–52.4)	53.6 (50.1–57.2)	52.8 (49.1–56.6)
Very low	47.9 (36.5–59.5)	44.1 (39.4–48.9)	46.6 (43.3–50)	46.6 (43.8–49.4)
***Bolsa Família***				
Yes	45.5 (39.3–51.9)	40.2 (34.6–46)	47.1 (42.9–51.3)	50.2 (47–53.4)
No	28.7 (24.4–33.3)	33.6 (29.2–38.3)	44.5 (41.2–47.9)	48.4 (45.3–51.6)
**Grandmother living in the household**				
Yes	34.1 (27.4–41.4)	41.2 (34.8–47.9)	49.6 (45.1–54.1)	45.6 (42.1–49.1)
No	28.7 (23.9–34.1)	33.4 (28.1–37)	43.4 (39.8–47)	50.6 (47.7–53.6)
**Family income (BRL)**				
<1,000.00	48.7 (43–54.4)	45.4 (36.2–54.8)	46.2 (41.7–50.9)	47.9 (44.7–51.1)
1,000.00 to 2,999.00	45 (34.5–56)	46.1 (40.3–52.1)	52.2 (48.9–55.4)	50.9 (47–54.9)
3,000.00 to 7,999.00	47.2 (39–55.6)	38.3 (31.6–45.6)	51.8 (46.9–56.7)	50.9 (44.3–57.5)
≥8,000.00	17.9 (12.7–24.6)	20 (15.2–25.7)	25.5 (19.3–47.7)	50.5 (32.3–68.5)
**Household crowding**				
Yes	30.5 (23.3–35.2)	34.4 (30.2–38.8)	45.7 (42.5–48.8)	50.8 (48.4–53.2)
No	24 (12.3–41.6)	39.2 (33.2–45.5)	38.7 (31.1–46.9)	38.9 (33.4–44.7)
**Private sector**				
Yes	14.7 (11–19.5)	18 (14–22.8)	28.3 (23.7–33.5)	42.4 (35–50.2)
No	49 (43.1–54.9)	46.7 (42.1–51.3)	51.2 (48.3–54)	49.8 (47.3–52.3)
**Difficulties in access**				
Yes	25.3 (16.5–36.7)	17.6 (11.8–25.6)	34.8 (28.1–42.2)	40.2 (33–47.7)
No	30.2 (25.8–35)	35.6 (31.4–40.1)	45.8 (42.8–48.8)	49.7 (47.3–52.2)
**Difficulties with the program**				
Yes	32.4 (25.7–39.8)	35 (29.2–41.2)	47.4 (42.8–52)	47.1 (43.4–50.9)
No	29 (24.4–34.2)	34.2 (29.5–39.2)	44.5 (41–48)	49.6 (46.9–52.2)
**Maternal characteristics**				
**Mother’s skin color**				
White	22.1 (17.6–27.5)	27.5 (22.1–33.7)	42.7 (38.2–47.4)	51.9 (47.7–55.9)
Black	63.6 (47.8–76.9)	49 (40.7–57.4)	48.8 (42–55.6)	53.4 (48.3–58.4)
Mixed-race	38.5 (32.4–44.9)	45.3 (40.5–50.1)	49 (45.7–52.2)	45.2 (42–48.5)
**Maternal age (years)**				
<20	24.3 (13.3–40.2)	19.2 (7.2–42)	51.8 (40.4–63)	41.9 (32.8–51.5)
20 to 34	41.8 (35.1–48.8)	44.5 (39.9–49.2)	47.7 (44.6–50.9)	48.1 (45.1–51.1)
35 and over	22.7 (17.8–28.4)	27.5 (22.8–32.7)	41.5 (36.9–46.3)	51.6 (47.6–55.6)
**Have a partner**				
Yes	28.4 (23.8–33.4)	32.9 (28.7–37.4)	45.2 (41.8–48.6)	50.1 (47.2–53)
No	44.9 (36.0–54.2)	43.1 (35.1–51.4)	45.7 (41.1–50.4)	47.1 (43.4–50.8)
**Mother’s work**				
Yes	28.8 (23.8–34.3)	29.7 (25.1–34.8)	44.1 (40.0–48.3)	51.2 (48.4–54)
No	34.1 (27.4–41.4)	42.9 (37.3–48.8)	47 (43.6–50.4)	47.1 (43.6–50.7)
**Maternal level of education (years)**				
**Up to eight**	29.6 (18.4–43.9)	32.8 (27.3–38.7)	46.2 (38.1–54.6)	43 (38.5–47.5)
**Nine to 12**	46.8 (32.4–61.7)	50.3 (43.0–57.5)	43.1 (37.5–48.9)	49.7 (44.6–54.7)
**13 to 15**	53.4 (45.2–61.5)	46 (40.2–52)	51.3 (48.5–63)	50.7 (47.4–54)
**16 and over**	21.8 (17.4–27)	27.3 (21.4–34)	39.6 (34.3–45.1)	48 (42.4–53.8)
**Child characteristics**				
**Child’s skin color**				
White	23.5 (18.8–28.9)	29.5 (24.7–34.9)	42.2 (38.1–46.3)	51 (47.4–54.7)
Black	63.2 (42.6–79.9)	36.9 (28.3–46.5)	53.1 (45.5–60.5)	50 (43.3–56.7)
Mixed-race	44.3 (36.9–52)	44.8 (39–50.7)	49.5 (46–53)	46.8 (30.9–66.5)
**Nursery or day care**				
Yes	30.1 (24.2–26.7)	28.5 (23.8–33.8)	43.6 (39.1–48.2)	52.1 (48.5–55.8)
No	29.8 (24.5–35.7)	41.3 (35.9–46.9)	46.5 (43.1–49.8)	46.2 (43.4–49.1)
**Birth order**				
Firstborn	29.4 (24.2–35.2)	31.2 (26–36.8)	45.7 (42.4–49.2)	51.4 (47.8–54.9)
Second-born	30.2 (24.1–37.1)	40.8 (34–47.9)	44.6 (39.9–49.5)	49 (45.3–52.7)
Third-born	34.4 (22.9–48.1)	35.8 (27.2–45.6)	49.4 (42.7–56.1)	46.8 (41.1–52.7)
Fourth-born and so on	24.1 (12.4–41.6)	29.2 (20.3–40)	31.3 (24–39.7)	43.1 (37.1–49.2)

For families in stratum B, we verified lower coverage in relation to the same previous variables, in addition to not living together with grandmothers and the frequency of children attending day cares or nurseries, besides difficulties in accessing the services. In stratum C, we perceive greater internal heterogeneity, with lower coverage observed for factors similar to those observed in stratum B.

In stratum D, the situation changes in relation to the other strata in various aspects. We found no differences in coverage for the level of consumption, income, level of education, and maternal age. Coverage is lower for children living together with grandmothers, mixed-race mothers, mothers who have no paid work, and children who are not enrolled in day care or nursery.

The occurrence of unvaccinated children was higher in stratum A (10.7%; 95%CI 8–14.2) and decreasing in the other strata, respectively: 7% (95%CI 5.4–9) in stratum B; 4.7% (95%CI 3.8–5.8) in stratum C; and 5.4% (95%CI 4.4–10.7) in stratum D.

In Table 3, we show the full immunization coverage and 95% confidence intervals as well as the relative risks and their intervals for the null model and the final model. Coverage was lower in strata A and B and higher in strata C and D, as shown by crude relative risks according to which there is a higher probability of full coverage for children living in strata C and D.

**Table 3:** Vaccination coverage by the full immunization schedule at 24 months of age, with valid doses, by socioeconomic strata of residence, and crude and adjusted relative risks — cohorts of 2017-2018. National Vaccination Coverage Survey, 2020.

Variable	Full coverage (95%CI)	Crude RR (95%CI)	Adjusted RR (95%CI)
**Null model**			
Socioeconomic stratum			
Stratum A	29.9 (25.8–34.4)	1	
Stratum B	34.5 (30.4–38.8)	1.15 (0.95–1.39)	
Stratum C	45.1 (42.2–50)	1.50 (1.28–1.76)	
Stratum D	49.2 (46.7–51.5)	1.64 (1.40–1.91)	
Final model			
Socioeconomic stratum			
Stratum A		1	1
Stratum B		1.15 (0.95–1.39)	1.05 (0.89–1.23)
Stratum C		1.50 (1.28–1.76)	1.26 (1.10–1.45)
Stratum D		1.64 (1.40–1.91)	1.29 (1.12–1.48)
Crowding (yes)			0.78 (0.69–0.88)
Birth order			
Firstborn			1
Second-born			0.96 (0.90–0.98)
Third-born			0.92 (0.83–1.03)
Fourth-born and so on			0.80 (0.70–0.91)
Difficulties in access (yes)			0.79 (0.69–0.90)
Exclusive use of public services (yes)			1.95 (1.71–2.23)
**Variable**	**Full coverage** **(95%CI)**	**Crude RR** **(95%CI)**	**Adjusted RR** **(95%CI)**
**Null model**			
Socioeconomic stratum			
Stratum A	29.9 (25.8–34.4)	1	
Stratum B	34.5 (30.4–38.8)	1.15 (0.95–1.39)	
Stratum C	45.1 (42.2–50)	1.50 (1.28–1.76)	
Stratum D	49.2 (46.7–51.5)	1.64 (1.40–1.91)	
**Final model**			
Socioeconomic stratum			
Stratum A		1	1
Stratum B		1.15 (0.95–1.39)	1.05 (0.89–1.23)
Stratum C		1.50 (1.28–1.76)	1.26 (1.10–1.45)
Stratum D		1.64 (1.40–1.91)	1.29 (1.12–1.48)
Crowding (yes)			0.78 (0.69–0.88)
Birth order			
Firstborn			1
Second-born			0.96 (0.90–0.98)
Third-born			0.92 (0.83–1.03)
Fourth-born and so on			0.80 (0.70–0.91)
Difficulties in access (yes)			0.79 (0.69–0.90)
Exclusive use of public service (yes)			1.95 (1.71–2.23)

95%CI: 95% confidence interval; RR: relative risk.

After adjusting the potential confounding variables, intra-household crowding, birth order, difficulty in accessing services, and exclusive use of public health services for vaccination remained significant, in addition to socioeconomic strata.

The differences between strata remain significant. Regardless of strata, intra-household crowding and difficulty in access reduce the probability of having full coverage, while the exclusive use of public services increases the probability of full coverage. The higher the birth order, the lower the probability of full coverage.

Income and level of education, for being intervening variables between socioeconomic strata and vaccine coverage, were not adjusted in the regression model, and data were stratified. In Table 4, we demonstrate the stratification of full immunization coverage and the relative risks for the variables family income and level of education, which showed an association between socioeconomic strata and coverage only when considering maternal level of education of 16 years and over and family income of BRL 8 thousand or more. The effect of higher maternal level of education is significant both in stratum C and D. We observed the same effect for family income of BRL 8 thousand or over in stratum D. Comparing the data presented in Table 3, coverage was slightly lower for children whose mothers had higher levels of education, except in strata C and D. The differences were even more evident for children whose families had higher incomes, except for strata D.

**Table 4:** Vaccination coverage by the full immunization schedule at 24 months of age, valid doses, and relative risk by socioeconomic strata of the residence area, stratified by high level of education and income. National Vaccination Coverage Survey, 2020.

	Maternal education 16 years and over	
**Strata**	Vaccination coverage	RR
**A**	21.8 (17.4–27)	1
**B**	27.3 (21.4–34)	1.24 (0.91–1.72)
**C**	39.5 (34.3–45.1)	1.81 (1.39–2.34)
**D**	48.0 (42.3–53.8)	2.19 (1.71–2.82)
	**Family income BRL 8 thousand and over**	
**Strata**	Vaccination coverage	RR
**A**	17.9 (12.7–24.6)	1
**B**	20 (15.2–25.7)	1.11 (0.73–1.70)
**C**	25.5 (19.3–32.9)	1.42 (0.93–2.17)
**D**	50.5 (32.2–68.6)	2.82 (1.70–4.65)

RR: relative risk.

## DISCUSSION

The main findings of the study were the low coverage observed in all strata, with the highest probability of the full vaccination schedule in children living in areas with the worst socioeconomic indicators (strata C and D). In addition to socioeconomic strata, the following factors were associated with full immunization coverage: birth order, intra-household crowding, and difficulty in accessing vaccination sites by some families. These three factors decreased the probability of full coverage from the second-born child onward. Conversely, the exclusive use of public services for vaccination increased the probability of full coverage.

Social inequalities in immunization coverage are present in different countries, both in high-income and middle- or low-income countries, varying in each context according to several factors related to residence areas and family, maternal, and child characteristics. Differences can be observed between strata and within each of them, often making it difficult to analyze the influences of a particular factor, as the behavior changes in each strata^6,7,8,9,10,11^.

In low-income countries, immunization coverage varies greatly, and inequalities tend to be more pronounced the lower the national coverage. Considering internal inequalities, coverage is generally lower among layers in the worst socioeconomic situation, although in countries with high national coverage there may be no significant differences between strata. Occasionally, there are countries with some level of inequality that may favor de poorest strata^8,9,10,11^.

This same trend was observed in Brazilian municipalities located in poorer regions. In the city of São Luís, state of Maranhão, in 2006, low coverage was verified with valid doses (61.8%) at 12 months of age and with a higher probability of incomplete immunization schedule for the poorest sections of the population^12^.

In Assis Brasil, state of Acre, in 2010, researchers showed an association between incomplete immunization schedule and worse socioeconomic conditions of families^13^. In Recife, state of Pernambuco, in 2015, authors of a study conducted in a favela with a population served by the Family Health program also recorded low coverage by the full immunization schedule and lower coverage among families in more precarious conditions^14^.

The sociodemographic factors of families, mothers, and children, which can be seen as mediators between the ecological strata and the observed immunization coverage, also vary according to the broader context. In a study conducted in Manchester, England, in areas with greater social deprivation, coverage for vaccines, such as the measles, mumps and rubella and pentavalent vaccines, reached high coverage that vary among ethnic groups, being more unfavorable to white families, corroborating the gradient observed for the general population. The authors emphasize that the role of socioeconomic deprivation depends on other predictors — such as access, cultural norms, and social support networks —, which also vary according to socioeconomic level^7^.

In the higher socioeconomic stratum, the lower vaccination coverage is related to higher level of consumption, higher monthly family income, higher maternal level of education, and mothers who self-reported to be white or mixed-race. However, for Black mothers, the coverage is higher if they live in stratum A. Unlike what was observed in Manchester and London, England, in areas with greater deprivation (stratum D), there were no differences in coverage according to ethnicity in this study^7^.

In Nepal, the coverage was higher the higher the family wealth quintile, and as coverage increased between 2001 and 2016, the differences between quintiles became progressively smaller, without changes in the gradient^8,11^. In Ethiopia, inequalities are pronounced among the wealth quintiles, favoring those in better conditions^9,11^.

In the city of Curitiba, state of Paraná (Brazil), in a cohort of 2002, no social inequalities were found in the coverage in relation to the full immunization schedule according to residence districts^15^. Authors of the survey conducted in São Luís, state of Maranhão (Brazil), in 2006, showed no differences in income, although levels of consumption C, D, and E were associated with a higher proportion of incomplete immunization schedule^12^. In an analysis of the live birth cohort in 2010, in the same city, researchers showed a disadvantage for families with levels of consumption D and E in the hierarchical analysis of predictors of the incomplete immunization schedule only for vaccines that were introduced more recently in the Brazilian National Immunization Program (PNI)^16^. In Assis Brasil, in the Amazon (Brazil), in 2010, incomplete coverage was associated with monthly income below USD 150, having lived in riverside areas, and not being a homeowner^13^.

In Araraquara, state of São Paulo (Brazil), for cohorts from 2014 to 2016, authors evaluated the impact of the *Bolsa Família* Program on immunization coverage. To establish an adequate comparison group, the authors used the propensity score pairing calculated from the following variables: mother without a partner, with one or more children, with less than seven prenatal appointments, Black or mixed-race ethnicity. The beneficiaries of this program showed high coverage at 12 months (92.1%) and at 24 months (83.8%) of age, with administered doses — and higher coverage than those of non-beneficiaries (85.1 and 73.6%) —, to meet the conditions established by the program. When considering only valid doses, coverage falls to 41.5 and 17% in beneficiaries and to 40.7 and 17.1% in controls, respectively, eliminating differences between groups^17^.

Authors of a study conducted in Nepal, with data from the demographic and health survey conducted in 2022, show a significant increase in coverage by the full immunization schedule (BCG+VOP3+DTP3+MR1) and a progressive reduction in inequalities between family wealth quintiles, corroborating the fact that, in higher coverage, inequalities tend to be reduced, even if the most privileged groups have the highest coverage^18^.

Maternal level of education was associated with full coverage in strata A, B, and C, with lower coverage in the most educated (16 years and over), without significant differences between strata A and B and significantly greater in strata C and D.

In Nepal, taking illiterate mothers as reference, coverage at 12 months of age were higher in all levels of education, showing the greatest difference after adjusting for wealth quintiles among mothers with secondary education (relative risk [RR]=3.4). Inequalities by maternal level of education were more pronounced than those observed by wealth quintiles based on family assets. The same pattern was observed in Ethiopia, but with smaller differences between levels of education than among wealth quintiles^8,9,10,11^.

In São Luís, state of Maranhão, there were no inequalities in relation to maternal level of education after adjustments for other variables, in both aforementioned surveys^12,15^. In Assis Brasil, maternal level of education less than eight years was associated with lower vaccination coverage as well as in the favela area in Recife^13,14^.

The birth order and household crowding, in turn, were significant factors when other variables were controlled. Decreasing coverage according to birth order was also observed in Ethiopia and São Luís, both for coverage with new and old vaccines^9,16^.

Access difficulties still persist, despite the expansion of the primary health care network and the implementation of the Family Health Strategy, involving several factors both in terms of structure and aspects more immediately linked to the families’ standards of living^19^.

The higher probability of attaining the full immunization schedule for children vaccinated exclusively in public health services, after adjustment for socioeconomic strata, crowding, birth order, and access difficulties, suggests that compliance with the vaccination schedule has been relatively more effective in public services.

One of the study limitations was the use of data from the 2010 Census for the definition of socioeconomic strata. After 10 years, the occupation of urban space may have changed in some areas. However, at the aggregate level, there is coherence between the strata of the residence area and the family indicators. The main advantages of this survey are the sample size and the possibility of investigating unvaccinated children, not relying on population estimates for the calculation of coverage and also being able to calculate valid doses from the data of the vaccination cards. The retrospective cohort design ensures the absence of loss to follow-up and the verification of full coverage for each child over the follow-up time.

Social inequalities in immunization coverage are evidenced both in high-income and in low- and middle-income countries, showing different distribution in each context, varying not only among countries, but also within them. Richer areas have a different pattern than those observed in the poorest areas, with different mediating factors and different impacts on each socioeconomic stratum. In this research, overall, vaccination coverage was lower in the highest strata and, within them, among the most privileged. For some of the studied factors, such as ethnicity, disadvantage situations could be mitigated for families living in the best areas (strata A and B). Within stratum D, although coverage was higher, the groups with worse standards of living had lower coverage, showing the effect of the accumulation of disadvantages on these families.

## SUPPLEMENTARY MATERIAL

The database is not yet available for public use.
