# Supplementary material for: Social inequalities and full vaccination coverage at 24 months – 2017-2018 live birth cohort: National Vaccination Coverage Survey, 2020
Source: Rev Bras Epidemiol. 2025 Nov 3;28:e250049. doi: 10.1590/1980-549720250049 (PMC12588615; doi:10.1590/1980-549720250049)
Supplement: Translation

# Translation: Desigualdades sociais da cobertura vacinal aos 24 meses – coorte de nascidos vivos em 2017-2018: Inquérito Nacional de Cobertura Vacinal, 2020

**DOI:** 10.1590/1980-549720250049.2

**Authors:** Rita Barradas Barata, Ana Paula França, Ione Aquemi Guibu, Carla Magda Alan Domingues, Maria da Gloria Teixeira, José Cássio de Moraes

**Affiliations:** I Faculdade de Ciências Médicas da Santa Casa de São Paulo, Departamento de Saúde Coletiva – São Paulo (SP), Brasil.; II Consultora – Brasília (DF), Brasil.; III Universidade Federal da Bahia, Instituto de Saúde Coletiva – Salvador (BA), Brasil.

**Keywords:** Cobertura vacinal, Fatores socioeconômicos, Estudos de coortes, Brasil

## Abstract

**Objetivo:**

Analisar desigualdades sociais na cobertura vacinal para o esquema completo aos 24 meses de idade, doses válidas, segundo estratos socioecômicos e características familiares, maternas e da criança.

**Métodos:**

Coorte retrospectiva de nascidos vivos em 2017–2018 nas capitais; no Distrito Federal; e em 12 cidades brasileiras, recrutadas em 2020–2021, acompanhadas a partir do nascimento por meio da caderneta de vacinação, sem perdas de seguimento. Os responsáveis foram entrevistados e as cadernetas foram fotografadas. Os setores censitários urbanos das 39 cidades foram agrupados em 4 estratos por análise de cluster da renda média, renda>20 sm, %alfabetizados. Em cada estrato foram sorteados o mesmo número de setores, e as crianças das coortes de interesse foram recrutadas até completar-se o tamanho da amostra. A cobertura pelo esquema completo foi calculada para cada criança considerando doses válidas (data e intervalo entre doses). As análises descritiva, estratificada e de regressões de Poisson foram realizadas no módulo survey do STATA 17.0.

**Resultados:**

Foram incluídas 37.801 crianças. Coberturas ficaram abaixo de 50% em todos os estratos, sendo menores nos estratos A e B. A probabilidade de ter o esquema completo foi menor a partir do segundo filho em famílias com aglomeração intradomiciliar e dificuldades de acesso aos serviços. O uso exclusivo de serviços públicos foi associado à maior cobertura completa.

**Conclusão:**

Coberturas vacinais foram menores nos estratos com melhores condições de vida (A/B). A cobertura vacinal segundo fatores familiares, maternos e individuais varia entre os estratos, não sendo possível estabelecer um padrão comum.

## INTRODUÇÃO

O Programa Nacional de Imunizações (PNI) foi criado em 1973 para prover acesso universal a um conjunto de vacinas visando o controle das doenças imunopreveníveis e a redução das taxas de mortalidade infantil. Porém só com a criação do Sistema Único de Saúde (SUS), em 1990, houve expansão significativa das unidades básicas de saúde, tornando universal o acesso à imunização gratuita em todo o país. Apesar da garantia de acesso universal, a cobertura vacinal é heterogênea segundo tipo de vacina, regiões, cidades, estratos socioeconômicos e outras variáveis relacionadas a características das crianças, das mães e das famílias^1,2,3^.

Inquérito Nacional realizado para a coorte de nascidos vivos em 2005 nas capitais e no Distrito Federal já apontava desigualdades sociais nas coberturas em algumas das cidades; e menores coberturas nos estratos sociais com melhores condições de vida em algumas delas ou coberturas menores nos estratos com piores condições de vida, retratando a heterogeneidade existente^3^.

Estudos de cobertura vacinal raramente analisam diferenças socioeconômicas por área de vizinhança utilizando doses válidas, limitando-se às doses aplicadas. Este estudo busca preencher essas lacunas.

O principal objetivo deste estudo foi analisar as desigualdades sociais na cobertura vacinal, esquema completo (CVEC), incluindo todas as vacinas do calendário nacional de vacinação até os 24 meses de idade, com doses válidas, de crianças nascidas em 2017 ou 2018, segundo estratos socioecômicos de área de residência e características familiares, maternas e da criança associadas à cobertura vacinal.

## MÉTODOS

Os dados utilizados neste artigo são parte do Inquérito Nacional de Cobertura Vacinal financiado pelo Ministério da Saúde, com o objetivo de avaliar a cobertura vacinal pelo esquema completo aos 24 meses^2^.

O desenho adotado foi coorte retrospectiva. As crianças nascidas em 2017–2018 foram incluídas com idade>24 meses, podendo assim apresentar o desfecho, não havendo perdas de seguimento. Cada criança foi acompanhada, retrospectivamente, por meio da caderneta de vacinação, da data de nascimento até completar 24 meses de idade. O desenho de coorte permite calcular as doses válidas aplicadas nas idades recomendadas e com os intervalos corretos entre doses, a partir do nascimento até os 24 meses, para cada criança da amostra e, assim, obter a cobertura vacinal completa com doses válidas (incidência cumulativa). O Quadro 1 apresenta o esquema completo e os critérios para definição das doses válidas. Os métodos utilizados no inquérito foram descritos com maior detalhamento em outra publicação^2^. Crianças sem caderneta foram consideradas não vacinadas após a busca negativa por registro no sistema de informações do PNI.

**Quadro 1.** Vacinas do esquema completo até 24 meses e critérios para doses válidas.

Vacina	Dose válida
BCG	aplicada em qualquer mês
Hepatite B	aplicada em qualquer mês
Pentavalente (DPT+Hib+HepB) 1ª dose	42 dias de vida ou mais
Pentavalente (DPT+Hib+HepB) 2ª dose	pelo menos 30 dias após a 1ª dose
Pentavalente (DPT+Hib+HepB) 3ª dose	pelo menos 30 dias após a 2ª dose
VIP 1ª dose	42 dias de vida ou mais
VIP 2ª dose	pelo menos 30 dias após a 1ª dose
VIP 3ª dose	pelo menos 30 dias após a 2ª dose
Rotavirus 1ª dose	45 a 180 dias de vida
Rotavirus 2ª dose	pelo menos 30 dias após a 1ª dose, até 180 dias
Meningo C 1ª dose	42 dias de vida ou mais
Meningo C 2ª dose	pelo menos 30 dias após a 1ª dose
Pneumo 1ª dose	42 dias de vida ou mais
Pneumo 2ª dose	pelo menos 30 dias após a 1ª dose
Tríplice viral 1ª dose	365 dias de vida ou mais
Tríplice viral 2ª dose	pelo menos 30 dias após a 1ª dose
Hepatite A	365 dias de vida ou mais
Varicela	452 dias de vida ou mais
Meningo C reforço	365 dias de vida ou mais
Pneumo reforço	365 dias de vida ou mais
VOP	365 dias de vida e 3 doses anteriores VIP
DPT	365 dias de vida ou mais

BCG: vacina de Calmette e Guerin; DPT: vacina de difteria, pertussis e tétano; Hib: vacina de Hemophilus influenza; HepB: vacina de hepatite B; VIP: vacina de vírus inativado da poliomielite; Meningo C: vacina de meningococco C; Pneumo: vacina de pneumococo; VOP: vacina oral de poliomielite.

Os estratos socioeconômicos foram definidos mediante análise de *cluster*, considerando todos os setores censitários urbanos — em cada capital, no Distrito Federal e em 12 cidades com mais de 100 mil habitantes não localizadas na região metropolitana, selecionadas em diferentes unidades da federação. As variáveis de estratificação foram: renda média dos responsáveis pelo domicílio, proporção de responsáveis alfabetizados e proporção dos responsáveis com renda mensal maior que 20 salários mínimos. Os dados foram obtidos do Censo Demográfico de 2010 do Instituto Brasileiro de Geografia e Estatística (IBGE)^4^, último disponível para o país no início da pesquisa.

O estrato A corresponde ao *cluster* dos setores com melhores indicadores socioeconômicos, seguindo-se os estratos B, C e D, todos representando as condições médias dos habitantes que residem nos setores censitários que os compõem.

Foram sorteados o mesmo número de conglomerados de setores, agrupados pelo número estimado de nascidos vivos em 2017–2018, em cada estrato. As crianças foram buscadas por meio dos endereços do Sistema de Informações sobre Nascidos Vivos (Sinasc) e por busca ativa nas áreas sorteadas. As perdas amostrais decorreram da não localização de crianças das coortes nos setores sorteados, uma vez que foi utilizado procedimento de amostragem inversa para evitar as recusas. Na amostra a perda foi de 6%, embora ela tenha sido diferencial para os estratos, sendo maior no estrato A^2^.

Os responsáveis pelas crianças foram entrevistados para obtenção dos dados familiares, das mães e das próprias crianças, e as cadernetas de vacinação foram fotografadas. As informações foram posteriormente incorporadas ao banco de dados após leitura cuidadosa por profissionais capacitados.

As características familiares foram: nível de consumo estabelecido pelo critério da Associação Brasileira de Empresas de Pesquisa^5^, participação no programa de transferência de renda Bolsa Família, avó morando junto, renda familiar mensal, e aglomeração domiciliar (mais de dois moradores por cômodo usado como dormitório). Já as características maternas se dividiram em: idade, presença de companheiro, trabalho remunerado, raça/cor autorreferida (IBGE), e escolaridade. Por sua vez, as características da criança foram: raça/cor (IBGE), sexo, ordem de nascimento, frequência a creche ou berçário, e possuir caderneta de vacinação.

Além dessas características, foram incluídos: uso de serviços privados para pelo menos uma vacina, decisão de vacinar dos responsáveis, dificuldades de acesso a serviços e dificuldades com funcionamento do programa (falta de insumos e ausência/atitudes dos profissionais).

As análises foram realizadas no programa STATA 17.0 utilizando o módulo *survey* para amostras complexas, com ponderação para perdas amostrais e calibração populacional. Foram calculadas as coberturas vacinais completas com doses válidas e IC95% em cada estrato, e as medidas de efeito na comparação entre os estratos foram obtidas por regressão de Poisson considerando o estrato A como referência. Aquelas variáveis associadas exclusivamente com os estratos foram testadas para interação, e as possíveis variáveis de confusão também foram analisadas. Permaneceram no modelo final de ajuste aquelas variáveis associadas à exposição e/ou ao desfecho que não eram intervenientes entre elas. O procedimento utilizado na regressão de Poisson foi o *backward stepwise,* tomando como referência o modelo nulo.

Para duas das variáveis intervenientes, renda e escolaridade, só foi observado efeito estatisticamente significante entre os estratos nas classes de maior valor, a saber escolaridade de 16 anos e mais e renda familiar de R$ 8 mil ou mais. A análise estratificada é apresentada.

O projeto foi aprovado pelo Comitê de Ética em pesquisa em seres humanos da Irmandade da Santa Casa de São Paulo (Parecer 4.380.019), e os participantes assinaram termos de consentimento livre e esclarecido.

### Disponibilidade de dados da pesquisa

O conjunto de dados que dá suporte aos resultados deste estudo não está disponível publicamente.

## RESULTADOS

A amostra estudada foi de 37.801 crianças nascidas em 2017–2018, distribuídas pelos estratos socioeconômicos, sendo 8.333 no estrato A, 9.418 no estrato B, 9.992 no estrato C e 10.058 no estrato D. Destas, 12,93% não tinham caderneta no estrato A; 11,86%, no estrato B; 4,80%, no estrato C; e 7,94%, no estrato D.

A Tabela 1 descreve a composição da amostra por estratos socioeconômicos com relação às características familiares, maternas e das crianças. No estrato A predominam famílias com nível de consumo alto e médio, menos de 10% de famílias com avós morando junto, renda familiar de R$ 8 mil ou mais, a maioria usando serviços privados para vacinação, e aproximadamente 1/5 relatando dificuldades com o programa de imunização. As mães são predominantemente brancas, com 35 anos e mais, quase 90% têm companheiros, e 73% trabalham. As crianças são predominantemente brancas, mais da metade frequentam berçários ou creches e são primeiros filhos.

**Table:** Tabela 1. Características familiares, maternas e das crianças em cada estrato socioeconômico da área de residência. Inquérito Nacional de Cobertura Vacinal, 2020.

Variáveis	Estrato A	Estrato B	Estrato C	Estrato D
**Características familiares**				
**Nível de consumo**				
Alto	20,8	8,9	3	0,4
Médio	50,5	41	31,4	8,7
Baixo	17,6	31,4	36,5	38,9
Muito baixo	11,1	18,7	29,1	52
**Bolsa Família**	7,3	13,9	20,5	34,5
**Avó morando junto**	22,4	24,1	27,4	30,4
**Renda familiar (R$)**				
<1.000,00	7	15,5	22,3	41,1
1.000,00 a 2.999,00	15,5	27,9	39,3	46,7
3.000,00 a 7.999,00	30,7	32,8	27,1	10,8
≥8.000,00	46,8	23,8	11,3	1,4
**Aglomeração domiciliar**	3,6	5,3	8,5	14,6
**Serviços privados**	56,3	43,4	26,8	9
**Decisão de vacinar (todas as vacinas)**	97,7	96,7	97,8	97,3
**Dificuldades de acesso**	5,7	7	5,4	8,2
**Dificuldades com o programa**	20,5	23,0	24,7	23,6
**Características maternas**				
**Cor da mãe**				
Branca	60,2	57,7	52,5	37,6
Preta	6,7	7,1	8,3	17,1
Parda	31,8	32,4	37,6	43,6
Outras (amarelas e indígenas)	1,3	2,8	1,6	1,7
**Idade materna (anos)**				
<20	0,8	2,1	1,6	3,1
20 a 34	37,7	42,5	54,2	62,7
35 e mais	61,5	55,4	44,2	34,2
**Companheiro**	88,9	83,3	78,4	71,4
**Trabalho materno remunerado**	72,6	63,6	58,1	47,9
**Características das crianças**				
**Cor da criança**				
Branca	69,7	62,9	59,3	45,2
Preta	2,6	4,4	4,7	10,8
Parda	26,5	31,5	35,1	42,8
Outras (amarela e indígena)	1,2	1,2	0,9	1,2
**Berçário ou creche**	56,9	52,9	48,6	48,5
**Ordem de nascimento**				
Primeiro	52,1	54,9	51,6	43,9
Segundo	35,4	30,7	32,7	29,8
Terceiro	8,9	10,1	10,9	15,1
Quarto e mais	3,6	4,3	4,8	11,2

No estrato B as principais diferenças com relação ao estrato A estão no nível de consumo e na renda um pouco menores, além do uso menos frequente dos serviços privados. No estrato C as famílias apresentam nível de consumo médio ou baixo, cerca de 20% são atendidas pelo Bolsa Família, 28% têm avós morando junto, pouco mais de 25% usam serviços privados de vacinação e relatam problemas com o programa. A maioria das mães são brancas, de 20 a 34 anos de idade, cerca de 78% têm companheiro, e a maioria tem trabalho remunerado. As crianças são majoritariamente brancas, mas menos da metade frequentam creches ou berçários, e metade são primeiros filhos.

No estrato D mais da metade das famílias tem nível de consumo muito baixo, 34% são beneficiárias do Bolsa Família, cerca de 30% têm as avós morando junto, 15% apresentam aglomeração domiciliar; e ¼ relatam problemas com o programa. As mães são predominantemente pardas, têm entre 20 e 34 anos de idade, 70% com companheiro, e menos da metade tem trabalho remunerado. As crianças são brancas ou pardas, menos da metade frequentam creches ou berçários, e são principalmente primeiros e segundos filhos.

Com relação à intenção de vacinar, a quase totalidade dos responsáveis afirmou que pretendia dar todas as vacinas aos filhos, independentemente do estrato socioeconômico.

A Tabela 2 apresenta as coberturas vacinais para o esquema completo aos 24 meses, em cada estrato socioeconômico, segundo características familiares, maternas e da criança. Para as famílias residentes no estrato socioeconômico A, coberturas menores foram observadas em nível de consumo alto ou médio, renda de R$ 8mil ou mais, uso de serviços privados para vacinar, mães brancas com <20 anos ou 35 anos e mais de idade, com companheiro, escolaridade de 16 anos e mais, e crianças brancas. Mas, como no estrato também residem algumas famílias pobres, para estas menores coberturas são observadas entre as sem direito à Bolsa Família e com escolaridade materna de até 8 anos.

**Tabela 2.** Cobertura vacinal pelo esquema completo aos 24 meses segundo características familiares, maternas e das crianças em cada estrato socioeconômico da área de residência. Inquérito Nacional de Cobertura Vacinal, 2020.

Variáveis	Estrato A	Estrato B	Estrato C	Estrato D
**Características familiares**				
**Nível de consumo**				
Alto	16,5 (9,9–26,4)	15,8 (7,5–30,1)	18,9 (11–30,5)	47 (13,2–83,7)
Médio	25,6 20,5–31,4)	26,9 (20,8–34)	37,6 (32,1–43,5)	47,5 (40,1–55)
Baixo	51,4 (42,4–60,3)	45,4 (38,7–52,4)	53,6 (50,1–57,2)	52,8 (49,1–56,6)
Muito baixo	47,9 (36,5–59,5)	44,1 (39,4–48,9)	46,6 (43,3–50)	46,6 (43,8–49,4)
**Bolsa Família**				
Sim	45,5 (39,3–51,9)	40,2 (34,6–46)	47,1 (42,9–51,3)	50,2 (47–53,4)
Não	28,7 (24,4–33,3)	33,6 (29,2–38,3)	44,5 (41,2–47,9)	48,4 (45,3–51,6)
**Avó morando junto**				
Sim	34,1 (27,4–41,4)	41,2 (34,8–47,9)	49,6 (45,1–54,1)	45,6 (42,1–49,1)
Não	28,7 (23,9–34,1)	33,4 (28,1–37)	43,4 (39,8–47)	50,6 (47,7–53,6)
**Renda familiar (R$)**				
<1.000,00	48,7 (43–54,4)	45,4 (36,2–54,8)	46,2 (41,7–50,9)	47,9 (44,7–51,1)
1.000,00 a 2.999,00	45 (34,5–56)	46,1 (40,3–52,1)	52,2 (48,9–55,4)	50,9 (47–54,9)
3.000,00 a 7.999,00	47,2 (39–55,6)	38,3 (31,6–45,6)	51,8 (46,9–56,7)	50,9 (44,3–57,5)
≥8.000,00	17,9 (12,7–24,6)	20 (15,2–25,7)	25,5 (19,3–47,7)	50,5 (32,3–68,5)
**Aglomeração domiciliar**				
Sim	30,5 (23,3–35,2)	34,4 (30,2–38,8)	45,7 (42,5–48,8)	50,8 (48,4–53,2)
Não	24 (12,3–41,6)	39,2 (33,2–45,5)	38,7 (31,1–46,9)	38,9 (33,4–44,7)
**Setor privado**				
Sim	14,7 (11–19,5)	18 (14–22,8)	28,3 (23,7–33,5)	42,4 (35–50,2)
Não	49 (43,1–54,9)	46,7 (42,1–51,3)	51,2 (48,3–54)	49,8 (47,3–52,3)
**Dificuldades de acesso**				
Sim	25,3 (16,5–36,7)	17,6 (11,8–25,6)	34,8 (28,1–42,2)	40,2 (33–47,7)
Não	30,2 (25,8–35)	35,6 (31,4–40,1)	45,8 (42,8–48,8)	49,7 (47,3–52,2)
**Dificuldades com o programa**				
Sim	32,4 (25,7–39,8)	35 (29,2–41,2)	47,4 (42,8–52)	47,1 (43,4–50,9)
Não	29 (24,4–34,2)	34,2 (29,5–39,2)	44,5 (41–48)	49,6 (46,9–52,2)
**Características maternas**				
**Cor da mãe**				
Branca	22,1 (17,6–27,5)	27,5 (22,1–33,7)	42,7 (38,2–47,4)	51,9 (47,7–55,9)
Preta	63,6 (47,8–76,9)	49 (40,7–57,4)	48,8 (42–55,6)	53,4 (48,3–58,4)
Parda	38,5 (32,4–44,9)	45,3 (40,5–50,1)	49 (45,7–52,2)	45,2 (42–48,5)
**Idade materna (anos)**				
<20	24,3 (13,3–40,2)	19,2 (7,2–42)	51,8 (40,4–63)	41,9 (32,8–51,5)
20 a 34	41,8 (35,1–48,8)	44,5 (39,9–49,2)	47,7 (44,6–50,9)	48,1 (45,1–51,1)
35 e mais	22,7 (17,8–28,4)	27,5 (22,8–32,7)	41,5 (36,9–46,3)	51,6 (47,6–55,6)
**Companheiro**				
Sim	28,4 (23,8–33,4)	32,9 (28,7–37,4)	45,2 (41,8–48,6)	50,1 (47,2–53)
Não	44,9 (36,0–54,2)	43,1 (35,1–51,4)	45,7 (41,1–50,4)	47,1 (43,4–50,8)
**Trabalho materno**				
Sim	28,8 (23,8–34,3)	29,7 (25,1–34,8)	44,1 (40,0–48,3)	51,2 (48,4–54)
Não	34,1 (27,4–41,4)	42,9 (37,3–48,8)	47 (43,6–50,4)	47,1 (43,6–50,7)
**Escolaridade materna (anos)**				
**Até 8**	29,6 (18,4–43,9)	32,8 (27,3–38,7)	46,2 (38,1–54,6)	43 (38,5–47,5)
**9 a 12**	46,8 (32,4–61,7)	50,3 (43,0–57,5)	43,1 (37,5–48,9)	49,7 (44,6–54,7)
**13 a 15**	53,4 (45,2–61,5)	46 (40,2–52)	51,3 (48,5–63)	50,7 (47,4–54)
**16 e mais**	21,8 (17,4–27)	27,3 (21,4–34)	39,6 (34,3–45,1)	48 (42,4–53,8)
**Características criança**				
**Cor da criança**				
Branca	23,5 (18,8–28,9)	29,5 (24,7–34,9)	42,2 (38,1–46,3)	51 (47,4–54,7)
Preta	63,2 (42,6–79,9)	36,9 (28,3–46,5)	53,1 (45,5–60,5)	50 (43,3–56,7)
Parda	44,3 (36,9–52)	44,8 (39–50,7)	49,5 (46–53)	46,8 (30,9–66,5)
**Berçário ou creche**				
Sim	30,1 (24,2–26,7)	28,5 (23,8–33,8)	43,6 (39,1–48,2)	52,1 (48,5–55,8)
Não	29,8 (24,5–35,7)	41,3 (35,9–46,9)	46,5 (43,1–49,8)	46,2 (43,4–49,1)
**Ordem de nascimento**				
Primeiro	29,4 (24,2–35,2)	31,2 (26–36,8)	45,7 (42,4–49,2)	51,4 (47,8–54,9)
Segundo	30,2 (24,1–37,1)	40,8 (34–47,9)	44,6 (39,9–49,5)	49 (45,3–52,7)
Terceiro	34,4 (22,9–48,1)	35,8 (27,2–45,6)	49,4 (42,7–56,1)	46,8 (41,1–52,7)
Quarto e mais	24,1 (12,4–41,6)	29,2 (20,3–40)	31,3 (24–39,7)	43,1 (37,1–49,2)

Para as famílias do estrato B, menores coberturas se repetem em relação a variáveis anteriores, sendo acrescidas ao fato de as avós não morarem junto e à frequência da criança a creche ou berçário, além de dificuldades de acesso aos serviços. No estrato C os resultados mostram maior heterogeneidade interna com menores coberturas observadas para fatores semelhantes aos observados no estrato B.

No estrato D a situação se modifica em relação aos outros estratos em vários aspectos. Não se observam diferenças nas coberturas para o nível de consumo, a renda, a escolaridade e a idade materna. A cobertura é menor para crianças com avó morando junto, mães pardas, sem trabalho remunerado, e que não frequentam creche ou berçário.

A ocorrência de criancas não vacinadas foi maior no estrato A (10,7%; IC95% 8–14,2) e decrescente nos demais estratos: respectivamente 7% (IC95% 5,4–9) no estrato B; 4,7% no (IC95% 3,8–5,8) estrato C; e 5,4% (IC95% 4,4–6,6) no estrato D

A Tabela 3 apresenta a cobertura completa e os intervalos de confiança de 95% bem como os riscos relativos e seus intervalos para o modelo nulo e para o modelo final. As coberturas foram menores nos estratos A e B e maiores nos estratos C e D, como mostram os riscos relativos brutos que apontam maior probabilidade de cobertura completa para criancas residentes nos estratos C e D.

**Tabela 3.** Cobertura vacinal pelo esquema completo aos 24 meses de idade, com doses válidas, por estratos socioeconômicos de residência, e riscos relativos brutos e ajustados — coortes de 2017-2018. Inquérito Nacional de Cobertura Vacinal, 2020.

Variável	Cobertura completa (IC95%)	RR bruto (IC95%)	RR ajustado (IC95%)
**Modelo nulo**			
Estrato socioeconômico			
Estrato A	29,9 (25,8–34,4)	1	
Estrato B	34,5 (30,4–38,8)	1,15 (0,95–1,39)	
Estrato C	45,1 (42,2–50)	1,50 (1,28–1,76)	
Estrato D	49,2 (46,7–51,5)	1,64 (1,40–1,91)	
**Modelo final**			
Estrato socioeconômico			
Estrato A		1	1
Estrato B		1,15 (0,95–1,39)	1,05 (0,89–1,23)
Estrato C		1,50 (1,28–1,76)	1,26 (1,10–1,45)
Estrato D		1,64 (1,40–1,91)	1,29 (1,12–1,48)
Aglomeração (sim)			0,78 (0,69–0,88)
Ordem de nascimento			
Primeiro			1
Segundo			0,96 (0,90–0,98)
Terceiro			0,92 (0,83–1,03)
Quarto e mais			0,80 (0,70–0,91)
Dificuldade de acesso (sim)			0,79 (0,69–0,90)
Serviço público exclusivo (sim)			1,95 (1,71–2,23)
**Variável**	**Cobertura completa** **(IC95%)**	**RR bruto** **(IC95%)**	**RR ajustado** **(IC95%)**
**Modelo nulo**			
Estrato socioeconômico			
Estrato A	29,9 (25,8–34,4)	1	
Estrato B	34,5 (30,4–38,8)	1,15 (0,95–1,39)	
Estrato C	45,1 (42,2–50)	1,50 (1,28–1,76)	
Estrato D	49,2 (46,7–51,5)	1,64 (1,40–1,91)	
**Modelo final**			
Estrato socioeconômico			
Estrato A		1	1
Estrato B		1,15 (0,95–1,39)	1,05 (0,89–1,23)
Estrato C		1,50 (1,28–1,76)	1,26 (1,10–1,45)
Estrato D		1,64 (1,40–1,91)	1,29 (1,12–1,48)
Aglomeração (sim)			0,78 (0,69–0,88)
Ordem de nascimento			
Primeiro			1
Segundo			0,96 (0,90–0,98)
Terceiro			0,92 (0,83–1,03)
Quarto e mais			0,80 (0,70–0,91)
Dificuldade de acesso (sim)			0,79 (0,69–0,90)
Serviço público exclusivo (sim)			1,95 (1,71–2,23)

IC95%: intervalo de confiança de 95%; RR: risco relativo.

Após o ajuste das potenciais variáveis de confusão, permaneceram significantes a aglomeração intradomiciliar, a ordem de nascimento, a dificuldade de acesso aos serviços e o uso exclusivo de serviços públicos de saúde para vacinação, além dos estratos socioeconômicos.

As diferenças entre os estratos permanecem significativas. Independentemente dos estratos, a aglomeração intradomiciliar e a dificuldade de acesso reduzem a probabilidade de ter a cobertura completa, enquanto o uso exclusivo de serviços públicos aumenta a probabilidade de ter cobertura completa. Quanto maior a ordem de nascimento, menor a probabilidade de cobertura completa.

A renda e a escolaridade, por serem variáveis intervenientes entre os estratos socioeconômicos e as coberturas vacinais, não foram ajustadas no modelo de regressão, e os dados foram estratificados. A Tabela 4 mostra a estratificação das coberturas completas e os riscos relativos para as variáveis renda familiar e escolaridade, que mostraram associação entre os estratos socioeconômicos e as coberturas apenas quando consideradas a escolaridade materna de 16 anos e mais e a renda familiar de R$ 8 mil ou mais. O efeito da escolaridade materna mais alta é significante tanto no estrato C quanto no D. O mesmo efeito se observa para a renda familiar de R$ 8 mil ou mais no estrato D. Comparando aos dados apresentados na Tabela 3, as coberturas foram um pouco menores para as crianças cujas mães tinham escolaridade mais alta, exceto nos estratos C e D. As diferenças foram ainda mais evidentes para as crianças cujas famílias tinham rendas mais altas, exceto no estrato D.

**Tabela 4.** Cobertura vacinal pelo esquema completo aos 24 meses, doses válidas e risco relativo por estratos socioeconômicos da área de residência, estratificada por escolaridade e renda altas. Inquérito Nacional de Cobertura Vacinal, 2020.

	Escolaridade materna 16 anos e mais	
**Estratos**	Cobertura vacinal	RR
**A**	21,8 (17,4–27)	1
**B**	27,3 (21,4–34)	1,24 (0,91–1,72)
**C**	39,5 (34,3–45,1)	1,81 (1,39–2,34)
**D**	48,0 (42,3–53,8)	2,19 (1,71–2,82)
	**Renda familiar R$ 8 mil e mais**	
**Estratos**	Cobertura Vacinal	RR
**A**	17,9 (12,7–24,6)	1
**B**	20 (15,2–25,7)	1,11 (0,73–1,70)
**C**	25,5 (19,3–32,9)	1,42 (0,93–2,17)
**D**	50,5 (32,2–68,6)	2,82 (1,70–4,65)

RR: risco relativo.

## DISCUSSÃO

Os principais achados do estudo foram as baixas coberturas observadas em todos os estratos, com maior probabilidade do esquema completo nas crianças residentes nas áreas com piores indicadores socioeconômicos (estratos C e D). Os fatores associados à cobertura completa, além dos estratos socioeconômicos, foram a ordem de nascimento, aglomeração intradomiciliar e a dificuldade de acesso aos locais de vacinação por algumas famílias. Estes três fatores diminuíram a probabilidade de cobertura completa, a partir do segundo filho. Já o uso exclusivo de serviços públicos para vacinação aumentou a probabilidade de cobertura completa.

As desigualdades sociais nas coberturas vacinais estão presentes em diferentes países, tanto naqueles de rendas altas quanto nos de renda média ou baixa, variando em cada contexto de acordo com diversos fatores relacionados às áreas de residência e características familiares, maternas e da criança. As diferenças podem ser observadas entre estratos e no interior de cada um deles, tornando muitas vezes difícil a análise das influências de um fator em particular, já que o comportamento é modificado em cada um dos estratos^6,7,8,9,10,11^.

Nos países de renda baixa as coberturas vacinais variam bastante, e as desigualdades tendem a ser mais acentuadas quanto menor for a cobertura nacional. Considerando as desigualdades internas, geralmente as coberturas são menores entre as camadas em pior situação socioeconômica, embora em países com cobertura nacional alta possa não haver diferenças significantes entre os estratos. Eventualmente, há países com alguma desigualdade em favor dos mais pobres^8,9,10,11^.

Essa mesma tendência foi observada em municípios brasileiros situados em regiões mais pobres. Em São Luís do Maranhão, em 2006, foram encontradas coberturas baixas com doses válidas (61,8%) aos 12 meses de idade e com maior probabilidade de esquemas incompletos para as camadas mais pobres de população^12^.

Em Assis Brasil, no estado do Acre, em 2010, estudo mostrou associação de esquemas incompletos com piores condições socioeconômicas das famílias^13^. Em Recife, 2015, estudo em uma favela com população atendida pelo programa Saúde da Família também registrou cobertura baixa pelo esquema completo e menor entre famílias com condições mais precárias^14^.

Os fatores sociodemográficos das famílias, mães e crianças, que podem ser vistos como mediadores entre os estratos ecológicos e as coberturas observadas, também variam em função do contexto mais amplo. Em estudo realizado em Manchester, em áreas com maior privação social, as coberturas para vacinas como a tríplice viral e a pentavalente alcançaram coberturas altas que variam entre os grupos étnicos, sendo mais desfavorável a famílias brancas, mantendo o gradiente observado para a população em geral. Os autores ressaltam que o papel das privações socioeconômicas é dependente de outros preditores como acesso, normas culturais e redes de apoio social — que também variam de acordo com o nível socioeconômico^7^.

No estrato socioeconômico mais alto a menor cobertura vacinal está relacionada a maior nível de consumo, maior renda familiar mensal, maior escolaridade materna e mães que se autodeclararam brancas ou pardas. Entretanto, para as mães pretas a cobertura é maior se elas residirem no estrato A. Ao contrário do que foi observado em Manchester e Londres, na Inglaterra, nas áreas com maior privação (estrato D) não houve diferenças de cobertura segundo etnia neste estudo^7^.

No Nepal as coberturas foram mais altas quanto maior o quintil de riqueza das famílias, e à medida em que a cobertura aumentou, entre 2001 e 2016, as diferenças entre os quintis tornaram-se progressivamente menores, sem que o gradiente tenha se modificado^8,11^. Na Etiópia as desigualdades são pronunciadas entre os quintis de riqueza, favorecendo aqueles em melhor situação^9,11^.

Em Curitiba, em coorte de 2002, não foram encontradas desigualdades sociais nas coberturas pelo esquema completo segundo distritos de residência^15^. O inquérito realizado em São Luís em 2006 não mostrou diferenças quanto à renda, embora os níveis de consumo C, D e E tenham sido associados com maior proporção de esquemas incompletos^12^. Análise da coorte de nascidos vivos em 2010, na mesma cidade, mostrou desvantagem para as famílias com nível de consumo D e E na análise hierárquica de preditores do esquema incompleto apenas para vacinas que foram introduzidas mais recentemente no PNI^16^. Em Assis Brasil, na Amazônia, em 2010 a cobertura incompleta estava associada a renda mensal abaixo de US$ 150, ter morado em áreas ribeirinhas e não ter a propriedade do domicílio^13^.

Em Araraquara, para coortes de 2014 a 2016, foi possível avaliar o impacto do Programa Bolsa Família na cobertura vacinal. Para estabelecer grupo de comparação adequado os autores utilizaram o pareamento por *propensity score* calculado a partir das seguintes variáveis: mãe sem companheiro, com um ou mais filhos, com menos de sete consultas de pré-natal, etnia preta ou parda. Os beneficiários do referido programa apresentaram coberturas altas aos 12 meses (92,1%) e aos 24 meses (83,8%), com doses aplicadas — e maiores do que as dos não beneficiários (85,1 e 73,6%) —, para atender à condicionalidade estabelecida pelo programa. Quando se consideram apenas as doses válidas, as coberturas caem para 41,5 e 17% nos beneficiários e 40,7 e 17,1% nos controles, respectivamente, eliminando as diferenças entre os grupos^17^.

Estudo realizado no Nepal com dados do inquérito de demografia e saúde realizado em 2022 mostra aumento significativo de cobertura pelo esquema completo (BCG+VOP3+DTP3+MR1) e redução progressiva das desigualdades entre os quintis de riqueza das famílias, confirmando a observação de que em coberturas mais altas as desigualdades tendem a se reduzir, ainda que os grupos mais privilegiados tenham as maiores coberturas^18^.

A escolaridade materna esteve associada à cobertura completa nos estratos A, B e C, produzindo coberturas menores nas mais escolarizadas (16 anos e mais), sem diferenças significantes entre estratos A e B e significantemente maior nos estratos C e D.

No Nepal, tomando como referências as mães sem escolaridade, as coberturas aos 12 meses foram maiores em todas as faixas de escolaridade, mostrando a maior diferença após o ajuste pelos quintis de riqueza entre as mães com educação secundária (RR=3,4). As desigualdades por escolaridade materna foram mais acentuadas do que as observadas por quintis de riqueza baseados nos bens familiares. O mesmo padrão foi observado na Etiópia, porém com diferenças menores entre os níveis de educação do que entre os quintis de riqueza^8,9,10,11^.

Em São Luís do Maranhão não foram observadas desigualdades com relação à escolaridade materna após os ajustes por outras variáveis, em ambos os inquéritos já mencionados^12,15^. Em Assis Brasil, escolaridade materna menor que 8 anos foi associada a menor cobertura vacinal, assim como na área de favela no Recife^13,14^.

A ordem de nascimento e a aglomeração domiciliar, por sua vez, mostraram-se fatores significantes quando outras variáveis foram controladas. Coberturas decrescentes segundo a ordem de nascimento também foram observadas na Etiópia e em São Luís do Maranhão, tanto para a cobertura com novas quanto com as antigas vacinas^9,16^.

As dificuldades de acesso ainda persistem, apesar da expansão da rede básica de serviços de saúde e da implementação da Estratégia Saúde da Família, envolvendo diversos fatores tanto de ordem estrutural quanto aspectos mais imediatamente ligados às condições de vida das famílias^19^.

A maior probabilidade de estar com o esquema completo para as crianças que foram vacinadas exclusivamente nos serviços públicos de saúde, após o ajuste pelos estratos socioeconômicos, por aglomeração, ordem de nascimento e dificuldades de acesso, sugere que nos serviços públicos o cumprimento do calendário vacinal tem sido relativamente mais efetivo.

Uma das limitações do estudo foi a utilização de dados do Censo 2010 para a definição dos estratos socioeconômicos. Passados 10 anos a ocupação do espaço urbano em algumas áreas pode ter se alterado. Entretanto, no nível agregado, há coerência entre os estratos de área de residência e os indicadores das famílias. As maiores vantagens deste inquérito são o tamanho da amostra e a possibilidade de investigar as criancas não vacinadas, não depender de estimativas populacionais para o cálculo da cobertura e, também, poder calcular doses válidas a partir dos dados das cadernetas. O desenho de coorte retrospectiva garante a ausência de perdas de seguimento e a verificação da cobertura completa para cada criança ao longo do tempo de seguimento.

As desigualdades sociais nas coberturas vacinais são evidenciadas tanto nos países de alta renda quanto nos de baixa e média, apresentando distribuição diferente em cada contexto, variando não apenas entre países, mas também dentro deles. Áreas mais ricas apresentam padrão diferente do observado nas áreas mais pobres, com fatores mediadores diferentes e com distintos impactos em cada estrato socioeconômico. Nesta pesquisa, de modo geral, as coberturas vacinais foram menores nos estratos mais altos e dentro deles, entre os mais privilegiados. Para alguns dos fatores estudados, como etnia, as situações de desvantagem podiam ser atenuadas para famílias residindo nas áreas melhores (estratos A e B). No interior do estrato D, embora a cobertura fosse mais alta, os grupos em piores condições tiveram coberturas menores, mostrando o efeito do acúmulo de desvantagens para essas famílias.

## MATERIAL SUPLEMENTAR

O banco de dados ainda não está disponivel para uso público.

## COMITÊ DE ÉTICA

Irmandade da Santa Casa de São Paulo, parecer 4.380.019
